# Mass drug administration significantly reduces infection of *Schistosoma mansoni *and hookworm in school children in the national control program in Sierra Leone

**DOI:** 10.1186/1471-2334-12-16

**Published:** 2012-01-22

**Authors:** Mary H Hodges, Nsa Dada, Anna Warmsley, Jusufu Paye, Momodu M Bangura, Emanuel Nyorkor, Mustapha Sonnie, Yaobi Zhang

**Affiliations:** 1 Helen Keller International, PO Box 369, Freetown, Sierra Leone; 2 Liverpool School of Tropical Medicine, Liverpool, UK; 3 National NTDCP, Ministry for Health and Sanitation, Freetown, Sierra Leone; 4 School of Community Health and Clinical Sciences, Njala University, Bo, Sierra Leone; 5 Helen Keller International, Regional Office for Africa, Dakar, Senegal

## Abstract

**Background:**

The first-ever round of school-based mass drug administration (MDA) with praziquantel together with mebendazole targeting school-aged children in endemic districts was conducted in 2009 by the National Neglected Tropical Diseases Control Program. To evaluate the impact of the treatment regimen, a cross-sectional sentinel site survey was conducted 6 months post-MDA.

**Methods:**

Fifteen sentinel schools from six highly endemic districts (according to data from national and pre-MDA surveys) with *Schistosoma mansoni *affecting over 50% of the population, and moderate to high prevalence of hookworms (> 20%). Approximately 30 children aged 9-14 years were selected from each school and stool samples (one per student) were examined by the Kato-Katz method.

**Results:**

The overall prevalence (and intensity) in these sentinel sites pre-MDA of *S. mansoni *was 69.0% (170.8 epg), hookworm: 41.7% (71.7 epg), *Ascaris lumbricoides: 1.8% *and *Trichuris trichiura: 3.8%*. Six months post MDA, the findings were *S. mansoni*: 38.2% (47.3 epg) and hookworm: 14.5% (8.7 epg), representing a reduction from pre-MDA levels of 44.6% (65.2%) and 72.3% (87.9%) respectively. The proportion of children who were moderately or heavily infected with *S. mansoni *fell from 35.6% pre MDA to 9.9% post MDA.

**Conclusions:**

Significant reduction in *S. mansoni *and hookworm infection was achieved by this first round MDA in school-going children in Sierra Leone. This reduction in infection burden can potentially contribute to a reduction of morbidity, such as anaemia, in these children.

## Background

Schistosomiasis and soil-transmitted helminthiasis (STH) inflict a significant health and socioeconomic burden in the tropics and subtropics particularly in sub-Saharan Africa [1-3]. It is estimated that over 200 million people are infected with schistosomes worldwide and about two billion with STHs [2,3]. Combined, schistosomiasis and STHs cause loss of 44 million or more disability adjusted life years (DALYs) each year, more than those due to malaria: 36 million and approaching due to tuberculosis: 47 million [4-6]. Schistosomiasis alone could be responsible for 200,000 deaths annually in sub-Saharan Africa, and STH responsible for 135,000 deaths per year globally [7].

Worm infections in children can result in stunting, wasting, diminished physical fitness, impaired memory and cognition [5,8] which combine to reduce educational performance, school attendance, future adult productivity, wage-earning capacity [5,9] and thus Gross National Productivity. These infections also have direct and indirect effects on malaria and HIV/AIDS in developing countries where they are co-endemic [10].

Sierra Leone is a country on the Atlantic coast in western Africa with a total population of about 6 million, of which approximately 25% are school-age children. Both *S. mansoni *and *S. haematobium *exist in the north-eastern parts of the country [11], and *S. mansoni *is the main species with focal prevalence of up to 95% [12-14]. National mapping and supplementary mapping of *S. mansoni *and STHs was conducted in 2008 and 2009 and spatial analysis predicted that *S. mansoni *was highly prevalent in the northeast [15,16]. Data on STH prevalence from the 1990s showed the national endemicity with ranges in school aged children for hookworm: 25-43%, *A.lumbricoides*: 32-93% and *T. trichiura*: 39-75% [17-22].

Annual MDA for STH commenced in some primary schools from 2004, biannual MDA in pre-school children from 2006 and annual MDA in the lymphatic filariasis elimination program from 2008.

The World Bank listed regular de-worming of children as one of the most cost-effective health interventions a developing nation can undertake and the World Health Assembly Resolution 54:9 encouraged member states to reach at least 75% of school aged children with regular annual de-worming in moderate-high transmission zones by 2010. In Sierra Leone, an integrated national control program on the neglected tropical diseases started in 2008 with financial support from the United States Agency for International Development and technical assistance from Helen Keller International. Following the disease mapping described above, a school-based MDA with praziquantel for schistosomiasis was performed for the first time in June 2009 in districts moderately-highly endemic for *S.mansoni *and mebendazole was also included in the distribution for STH. No schistosomiasis control activities had taken place prior to this first round of MDA in 2009.

This study reports the prevalence and intensity of *S. masoni *and STH in school children 9-14 years of age in these endemic sentinel sites 6 months after treatment with praziquantel and mebendazole and compares these with the pre-MDA levels. The paper reports on the protocols put in place prior to MDA to alleviate potential side effects and the additional costs these represent to the program.

## Methods

### National control program

A National Neglected Tropical Disease Control Program (NTDCP) was established in Sierra Leone by the Ministry of Health and Sanitation in 2008 to target onchocerciasis, lymphatic filariasis, schistosomiasis, STH and trachoma. It was based upon the existing Community Directed Treatment with Ivermectin (CDTI) platform developed for onchocerciasis control [23]. As part of the integrated effort for NTD control, national mapping of schistosomaisis and STH was conducted in 2008 [15] and a complementary survey in 6 highly endemic districts in 2009 [16]. Following the mapping, MDA activities were planned by the NTDCP. Considering the high prevalence of schistosomiasis in many districts and the first ever large-scale praziquantel distribution in the country, the MDA was started in a carefully planned, phased manner. The first phase targeted only school-going children in all primary schools in six highly or moderately endemic districts in June 2009 through school-based drug delivery. To minimize the side-effects of praziquantel treatment in this first-ever MDA, a special school feeding program for children to be treated was arranged with the support of USAID, as in rural settings in Sierra Leone, many children go to school in the morning without a meal and some may not have eaten the night before. The health workers were trained not to give praziquantel to any child with a history of convulsions during MDA. Supplies of anti-histamines and paracetamol were donated to the NTDCP by the St Andrews Clinic for Children-SL a local NGO, for distribution by health staff to children if any mild side-effects might occur. A total of 562,980 school children were treated with praziquantel and 549,701 were also treated with mebendazole with reported coverage of 94% and 92% respectively in this first round of MDA [23].

### Study site selection and data collection

From the mapping in 2008 and the complementary survey in 2009, fifteen (15) schools shown to have high prevalence of schistosomaisis (≥ 50%) and moderate or high prevalence of hookworm (≥ 20%) were selected as sentinel sites from Bo (Southern Province), Kailahun, Kenema and Kono (Eastern Province), Tonkolili and Koinadugu (Northern Province). Data collection for pre-MDA was already described previously [15,16]. Briefly, a single stool sample was collected from each participant and one slide per sample was prepared and examined using the Kato-Katz method by experienced examiners.

Six months after the treatment with praziquantel and mebendazole a cross-sectional survey was conducted in the selected sentinel schools. After sensitization of the community and school teachers approximately 30 children aged 9-14 years were randomly sampled in each site without discrimination by gender, religion or ethnicity. A single stool sample was collected from each participant, labelled and preserved in 10% formalin as did previously [16]. Participants were given albendazole 400 mg after the collection of specimens. The samples were transported back and later examined in the Njala University laboratory using the Kato-Katz method using a 41.7 um template (Vestergard Asia, PVT Ltd, India). One slide per sample was prepared and examined by experienced examiners. *S. mansoni *and STH eggs were counted and intensity of individual infections were calculated and expressed as eggs per gram of faeces (epg). Infections in children were classified as low, moderate or heavy infections according to the intensity of infection based upon the World Health Organization classifications [24].

### Data analysis

Results obtained were entered into Epi info^® ^version 3.5.1 and exported into SPSS 19.0 for analysis. For data from the surveys before MDA only those children aged 9-14 years were included in the current analysis. Analysis was done using SPSS Complex Samples (districts as strata and schools as clusters). Samples in each school were weighted according to the standard sample size (30). Chi square test was used to compare the prevalence before and after MDA. Kruskal Wallis test was used to compare the intensity of infection before and after MDA. The 95% confidence intervals (CIs) for prevalence were calculated using the Wilson score method without continuity correction after adjusting for sample weighting [25]. GPS coordinates were recorded and ArcMap 9.2 software was used to plot school locations.

### Ethical approval

Ethical approval for the survey was obtained from the Ministry of Health and Sanitation, Sierra Leone and the Liverpool School of Tropical Medicine Research Ethics Committee. Community informed consent was obtained following discussion with District Medical Officers, Chiefdom school inspectors, head teachers and community-teachers associations. Individual verbal consent was obtained from parents or carers as literacy rates among parents were low in most of these locations.

## Results

A total of 448 children were examined 6 months after the MDA. There was no significant difference in the sample size by sex: boys 227, girls 221.

### *Schistosoma mansoni*

**Figure 1:**
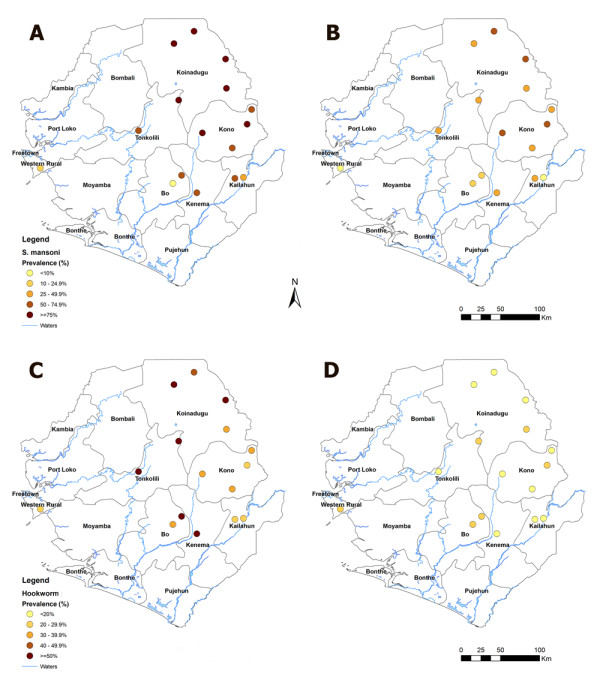
**Mapping of sites and prevalence of *S mansoni *and hookworm before and after MDA**.

The overall prevalence of *S. mansoni *infection in school children aged 9-14 years in these sentinel sites was 69.0% (95% CI: 64.7-73.2%) before MDA and it was 38.2% (95% CI: 33.8-42.8%) 6 months after MDA as shown in Table 1 and illustrated in Figure 1. This represents a significant reduction of 44.6% in prevalence after a single dose of praziquantel (*p *< 0.001). The overall intensity of *S. mansoni *infection was 170.8 epg (95% CI: 84.2-257.3 epg) before MDA and it was 47.3 epg (95% CI: 11.2-83.4 epg) 6 months after MDA. This represents a significant reduction of 72.3% from moderate to low mean intensity of infection (*p *< 0.001). Most importantly, the proportion of moderately and heavily infected children decreased from over 35.6% to 9.9% (Table 2).

**Table 1:** Prevalence and intensity of *S mansoni *and hookworm in children aged 9-14 years before and 6 months after praziquantel and mebendazole administration by district and by sex

	Pre-MDA			Post-MDA			Reduction (%)	
	
	No of children examined	*S.mansoni*	Hookworm	No of Children examined	*S.mansoni*	Hookworm	*S.mansoni*	Hookworm
**Prevalence (%)**								
Overall	515	69.0 (64.7-73.2)	41.7 (37.3-46.4)	448	38.2 (33.8-42.8)	14.5 (11.6-18.1)	44.6	65.2
By district								
Bo	60	33.3 (22.7-45.9)	48.3 (36.2-60.7)	60	21.7 (13.1-33.6)	26.7 (17.1-39.0)	34.8	44.7
Kailahun	60	56.7 (44.1-68.4)	21.7 (13.1-33.6)	60	25.0 (15.8-37.2)	11.7 (5.8-22.2)	55.9	46.1
Kenema	38	73.7 (55.6-85.8)	50.0 (15.8-37.2)	29	41.4 (25.5-59.3)	10.3 (3.6-26.4)	43.8	79.4
Koinadugu	177	82.9 (75.7-88.9)	47.3 (38.8-56.4)	120	45.0 (36.4-53.9)	12.5 (7.7-19.6)	45.7	73.6
Kono	120	75.0 (66.6-81.9)	32.5 (24.8-41.3)	119	50.4 (41.8-59.3)	10.9 (6.5-17.8)	32.8	66.5
Tonkolili	60	75.0 (62.8-84.2)	58.3 (45.7-69.9)	60	28.3 (18.5-40.8)	18.3 (10.6-29.9)	62.3	68.6
By sex								
Boys	262	69.4 (63.2-75.0)	50.8 (44.2-57.1)	227	40.1 (33.9-46.6)	19.4 (14.8-25.0)	42.2	61.8
Girls	252	68.9 (62.7-74.8)	32.5 (26.9-39.2)	221	36.2 (30.2-42.7)	9.5 (6.3-14.1)	47.5	70.8
**Intensity of infection (epg)**								
Overall	515	170.8 (84.2-257.3)	71.7 (44.1-99.3)	448	47.3 (11.2-83.4)	8.7 (3.5-14.0)	72.3	87.9
By district								
Bo	60	36.0 (0-114.4)	84.4 (31.8-137.0)	60	6.4 (2.8-10.0)	13.6 (4.7-22.5)	82.2	83.9
Kailahun	60	53.6 (32.2-75.0)	14.4 (0-32.2)	60	22.1 (0-44.6)	4.0 (2.2-5.8)	58.8	72.2
Kenema	38	111.8 (61.4-162.2)	79.6 (42.9-116.2)	29	31.4 (6.1-56.8)	4.1 (0-9.6)	71.9	94.8
Koinadugu	177	172.5 (133.8-211.2)	107.3 (9.9-204.7)	120	55.8 (13.2-98.4)	8.6 (0-22.0)	67.7	92.0
Kono	120	326.6 (14.3-638.9)	28.0 (21.1-34.9)	119	86.9 (0-214.2)	8.1 (0-17.9)	73.4	71.1
Tonkolili	60	137.2 (0.8-273.6)	128.4 (116.8-140.0)	60	25.6 (0-55.9)	12.4 (0-31.1)	81.3	90.3
By sex								
Boys	262	165.4 (90.9-239.9)	89.5 (45.1-133.9)	227	57.1 (4.8-109.4)	14.1 (3.9-24.3)	65.5	84.2
Girls	252	177.2 (73.7-280.6)	53.5 (32.6-74.5)	221	37.2 (15.8-58.7)	3.3 (1.4-5.2)	79.0	93.8

**Table 2:** Percentage proportions of children infected with *S. mansoni *before and after MDA

Intensity of infection	Proportion (95% CI) before MDA (%)	Proportion (95% CI) after MDA (%)
0 epg	29.5 (25.7-33.6)	56.8 (52.3-61.1)
Light 1-99 epg	35.0 (31.0-39.2)	25.2 (21.6-29.2)
Moderate 100-399 epg	24.1 (20.6-28.0)	7.8 (5.7-10.5)
Heavy ≥ 400 epg	11.5 (9.0-14.5)	2.1 (1.1-3.7)

Both prevalence and intensity of *S. mansoni *infection showed noticeable reduction across the districts surveyed after a single praziquantel MDA, with reduction rate ranging from 32.8% to 62.3% and 58.8-82.2% respectively. Among 15 sentinel schools, only four (4) schools still showed *S. mansoni *prevalence of over 50% after MDA, compared with 13 schools before MDA (details not shown). There was no significant difference in the of *S. mansoni *infection reduction by sex.

### Soil-transmitted helminths

The main STHs found in the surveys were hookworms, *A. lumbricoides *and *T. trichiura*. The overall prevalence of hookworm infection before MDA was 41.7% (95% CI: 37.3-46.4%), and it was 14.5% (95% CI: 11.6-18.1%) 6 months after MDA as shown in Table 1 and illustrated in Figure 1. This represents a significant reduction of 65.2% in prevalence (*p *< 0.001). The overall intensity of hookworm infection was 71.7 epg (95% CI: 44.1-99.3 epg) before MDA, and it was 8.7 epg (95% CI: 3.5-14.0 epg) 6 months after MDA, representing a significant reduction of 87.9% (*p *< 0.001). Such reduction in intensity of infection was seen across the districts surveyed. Among 15 sentinel schools, none of the schools showed prevalence of hookworm infection of over 50% after MDA, compared with 6 schools before MDA (details not shown).

As previously reported [15], the overall prevalence and intensity of *A. lumbricoides *and *T. trichiura *infections were low before MDA at 1.8% (95% CI: 1.0-3.5%) and 3.8% (95% CI: 2.4-5.9%) and 7.1% (95% CI: 5.2-9.8%) and 2.1% (95% CI: 1.1-3.8%) 6 months after MDA respectively. The overall intensity of *A. lumbricoides *and *T. trichiura *infections was 7.3 epg (95% CI: 0-15.7 epg) and 12.1 epg (95% CI: 0-27.6) before MDA, and was 6.0 epg (95% CI: 0-13.8 epg) and 1.2 epg (95% CI: 0-2.8 epg), respectively. Further analysis was not performed. There was no significant difference in the of Hookworm infection reduction by sex.

### Side effects

Despite the protocols in place of feeding children before praziquantel treatment, as many as 30% of children in some schools were feeling unwell following treatment, mostly with abdominal pain, nausea, vomiting, diarrhoea and dizziness (Turay, unpublished data). These were self limiting or responded to the donated drugs provided with only one case requiring an intravenous infusion and one case of convulsion though it was not clinically confirmed to be due to praziquantel treatment (NTDCP report).

### Costs

The total additional cost of delivering MDA in 2009 was 169,010 US dollars excluding the salaries from the Ministry of Health and Sanitation, logistic, drug supplies and infrastructure or 0.15 US dollars per child treated against schistosomiasis and STH. The total cost of performing the post MDA survey was 9,900 US dollars ($4.3 per child) including the costs of surveying 504 pre-school children aged 4-5 years old reported separately. In addition the costs of providing drugs to treat common side effects: paracetamol, oral rehydration salts and anti-histamines (donated) were estimated as 3,000 US dollars.

## Discussion

The national mapping data illustrated the significant burden caused by S. *mansoni *and hookworm to school-age children in Sierra Leone. A single round of praziquantel and mebendazole significantly reduced prevalence and intensity of both infections 6 months after treatment. Overall prevalence of *S. mansoni *fell from high to moderate and hookworm from moderate to low 6 months post MDA. Overall intensity of *S. mansoni *fell from moderate to low and hookworm intensity which was already low pre MDA fell significantly 6 months post MDA. Importantly the proportion of heavy infections was greatly reduced and this would certainly contribute to the reduction of morbidity, including anaemia, in school age children as shown previously by others [26,27]. The present results on *S. mansoni *from the national NTD control program in Sierra Leone are in line with the results from other national programs e.g. in Uganda [28,29]. According to the national mapping surveys in 2008-09, *S. haematobium *is also present in these districts (Hodges et al. unpublished data). *S. haematobium *was not monitored during sentinel site survey due to logistic reason, however, a significant reduction in *S. haematobium *infection is also anticipated, as it has been proved by others that a single praziquantel treatment significantly reduced both prevalence and intensity of *S. haematobium *infection in the large-scale national control programs in Niger and Burkina Faso [30,31].

A lesson has been learnt from Swaziland where de-worming with praziquantel caused serious political problem leading to the program suspension due to large number of children falling ill after taking the tablets [32]. In order to avoid such problems in the national control program in Sierra Leone, serious measures were taken to start the treatment. However the intensive community sensitisation both pre-and during implementation kept communities informed of potential side-effects and long-term benefits of the treatment. After gaining experience from this first MDA, the schistosomaisis control program was scaled up to include all school-age children and at risk adults in endemic districts in 2010. Communities understood the situation and showed high compliance when the second round of MDA was delivered. This provided an example for other countries to start their first round of large-scale praziquantel MDA, particularly in high endemicity areas.

There was a significant increase (*p *< 0.05) in *Ascaris *prevalence between pre and post-MDA results at two sites: Sulima in Koinadugu District (0% pre-MDA and 17% post-MDA) and Lei, in Kono District (0% pre-MDA and 20% post-MDA). The prevalence of hookworm and *T. trichiura *in Lei post MDA had not changed significantly: 27-23%, and 3-0% respectively. This may have been simply due to the sampling bias in two cross-sectional surveys. On the other hand, Sulima and Lei are both extremely 'Hard to Reach'. These results concur with a shortage of mebendazole supplies that were later reported by some health staff after MDA and are reflected in the numbers of children treated with praziquantel: 562,980 versus mebendazole: 549,701. Shortage of supplies cannot be easily corrected during MDA due to the rough terrain in 'Hard to Reach' locations and the commencement of the rainy season making transportation over roads and rivers treacherous. This illustrates how extremely vulnerable children in the 'Hard to Reach' areas are, still at risk of not receiving treatment even when a successful MDA campaign such as this achieved a reported coverage of 92% and overall satisfactory parasitological results.

Monitoring and evaluation of the impact is a crucial component of the national NTD control program. It is important to monitor the progress and to fine-tune a treatment strategy according to local changing epidemiological situations [31]. This area has been relatively less well funded within the current funding stream. Thanks to the UNICEF support, the current sentinel site survey was conducted and a further impact assessment survey of school-aged children in these same sentinel sites is being planned for pre-MDA in 2012. It is noted that in the current survey there was a strong representation of sites with high endemicity level (prevalence ≥ 50%). It would be useful to select some sites in the moderate and low endemicity categories to give a better understanding of overall progress of the national program in the future surveys. Another limitation of the study is the diagnostic method used. As discussed previously [16], the addition of formalin as a preservative may have diluted the stool samples and caused underestimation of the prevalence and potential overestimation of the intensity of infection. However, the sentinel sites for this study were in remote, 'hard to reach' locations where it was not possible to process and examine the stool samples on site. Adding formalin to preserve the stool samples was a necessary trade-off to avoid disintegration of helminth eggs, particularly hookworm, eggs. And during the preparation of the slides, care was taken to avoid access liquid. Considering the similar method was used both before and after MDA, such slight misestimating would not make much difference to the overall impact of the treatment shown here.

## Conclusions

This study showed that a significant impact on *S. mansoni *and hookworm infections was achieved in the first round of MDA with high coverage rates in Sierra Leone. This will have a beneficial effect on health, growth and school achievement.

## Competing interests

The authors declare that they have no competing interests.

## Authors' contributions

MH conceived, designed, supervised the study, drafted and revised the paper; MS and EN coordinated the field data collection; ND, AW, LB and JP performed the laboratory investigation and initial data analysis. JP and MH performed the initial data analysis. YZ conducted final data analysis and revised the paper. All authors reviewed the paper. All authors read and approved the final manuscript.

## Pre-publication history

The pre-publication history for this paper can be accessed here:

http://www.biomedcentral.com/1471-2334/12/16/prepub
